# LncRNA MIR99AHG mediated by FOXA1 modulates NOTCH2/Notch signaling pathway to accelerate pancreatic cancer through sponging miR-3129-5p and recruiting ELAVL1

**DOI:** 10.1186/s12935-021-02189-z

**Published:** 2021-12-15

**Authors:** Jin Xu, Weixue Xu, Xuan Yang, Zhen Liu, Yiya Zhao, Qinyun Sun

**Affiliations:** Department of General Surgery, Shengjing Hospital of China Medical University, No.36, Sanhao Street, Heping District, Shenyang, 110004 China

**Keywords:** Pancreatic cancer, MIR99AHG, ELAVL1, miR-3129-5p, NOTCH2

## Abstract

**Background:**

Pancreatic cancer (PCa) is a fatal malignancy with poor prognosis, high recurrence and mortality. Substantial reports have suggested long non-coding RNAs (lncRNAs) are implicated in development of numerous malignant tumors, and PCa is included. However, the correlation between novel lncRNA mir-99a-let-7c cluster host gene (MIR99AHG) and PCa remains elusive and needs to be deeply investigated.

**Methods:**

In this study, we firstly used RT-qPCR to examine MIR99AHG expression. Functional assays were implemented for determination of the role of MIR99AHG in PCa cells. Mechanism experiments were designed and carried out for exploring the regulatory mechanism involving MIR99AHG.

**Results:**

MIR99AHG was distinctly overexpressed in PCa cell lines. MIR99AHG deficiency abrogated PCa cell proliferation, migration and invasion. Moreover, MIR99AHG up-regulation was induced by transcription factor forkhead box A1 (FOXA1). Furthermore, MIR99AHG modulated notch receptor 2 (NOTCH2) expression and stimulated Notch signaling pathway through sequestering microRNA-3129-5p (miR-3129-5p) and recruiting ELAV like RNA binding protein 1 (ELAVL1).

**Conclusions:**

Altogether, the exploration of FOXA1/MIR99AHG/miR-3129-5p/ELAVL1/NOTCH2 axis in the progression of PCa might provide a meaningful revelation for PCa diagnosis and treatment.

**Supplementary Information:**

The online version contains supplementary material available at 10.1186/s12935-021-02189-z.

## Background

Pancreatic cancer (PCa) is defined to be a malignant tumor which occurs in the pancreas. It is commonly considered to be one of the most deadly solid tumors [1]. Nearly 277,000 new PCa cases are diagnosed worldwide annually, while 49,000 of them were from US and Europe [2]. In China, the occurrence and mortality of PCa is also increasing constantly [3]. Although many efforts have been made, the survival rate of PCa remains low. What’s more, the detection of PCa at early stage is difficult to achieve. Besides, treatment regiments at advanced stages are ineffective in most cases, as a result of which, the prognosis is very poor [4]. In addition, PCa has a poor response to most chemotherapeutic agents [5]. Therefore, it is imperative to discover the biological mechanisms which contribute to the occurrence and development of PCa.

Long non-coding RNAs (lncRNAs) are a class of transcripts with more than 200 nucleotides. Emerging evidence is demonstrating the critical role of lncRNAs in regulating gene expression, thereby exerting promoting or suppressive effects in the biological processes of cancers, including cell growth, apoptosis, migration, invasion and so on [6]. For example, lncRNA HOTAIR has been proposed to influence cell malignant behaviors via the miR-20a-5p/HMGA2 axis in breast cancer [7]. LncRNA DANCR directly targets miR-145 to accelerate tumor growth and angiogenesis in ovarian cancer [8]. LncRNA HOXA-AS2 facilitates tumorigenesis and development of papillary thyroid cancer by via miR-15a-5p/HOXA3 axis [9]. The significance of lncRNAs in PCa has also been widely verified [10]. For instance, it has been demonstrated that lncRNA ZEB2-AS1 sponges miR-203 and up-regulates HMGB1 expression to participate in the development of PCa [11]. Also, the XIST/miR-429/ZEB1 axis in PCa has also been validated [12]. Moreover, lncRNA TP73-AS1 serves as a sponge for miR-141-3p to boost the progression of PCa [13].

MIR99AHG has been reported to be involved in lung squamous cell carcinoma, head and neck squamous cell carcinoma and correlated with the survival rate [14, 15]. In addition, Meng et al. have proved that lncRNA MIR99AHG sequesters miR-577 and regulates FOXP1 expression to accelerate EMT and inhibit apoptosis of gastric cancer cells [16]. Nevertheless, the role of MIR99AHG in PCa has not gone through deep investigation. In this study, we paid concentrations to disclosing the function of MIR99AHG on the development of PCa and probing into the regulatory mechanism of MIR99AHG in PCa.

## Methods

### Cell culture

Four human pancreatic cancer cell lines (ASPC-1, BXPC-3, PANC-1 and SW1990) and normal immortalized human pancreatic epithelial cell line (HPDE6-C7) were selected out for this study. Among them, ASPC-1, BXPC-3, PANC-1 and SW1990 cell lines were all obtained from American Type Culture Collection (ATCC; Manassas, VA, USA) while HPDE6-C7 cell line was purchased from Huatuo Biotechnology Co., Ltd. (Shenzhen, China). ASPC-1, BXPC-3 cell lines were maintained in RPMI-1640 Medium. PANC-1 and HPDE6-C7 cell lines were cultured in Dulbecco’s Modified Eagle’s Medium (DMEM) (Invitrogen, Carlsbad, CA, USA). SW1990 cell line was cultured in Leibovitz's L-15 medium. All mediums were added with 10% fetal bovine serum (FBS) and cultured at 37 °C with 5% CO_2._

### Cell transfection

The short hairpin RNAs (shRNAs) targeting MIR99AHG, KLF4, FOXA1, ELAVL1 and relative controls were generated by GenePharma (Shanghai, China). In addition, pcDNA3.1 vectors were inserted with the sequences of KLF4, FOXA1 and NOTCH2 for overexpression. MiR-3129-5p mimics/inhibitor and negative control were procured from RiboBio (Shanghai, China). All cell transfections were conducted with Lipofectamine 2000 (Invitrogen, Carlsbad, CA, USA).

### Quantitative real-time PCR (RT-qPCR) analysis

According to the user’s manual, TRIzol Reagent (Introgen, Carlsbad, CA, USA) was utilized to obtain total RNA. Then, Reverse transcription of RNAs into cDNA was accomplished with the application of RevertAid First Strand cDNA Synthesis Kit (Thermo fisher, IL, USA). SYBR Green PCR Master Mix (Applied Biosystems, Foster City, CA, USA) was used to quantify RNA levels. Expression of the detected genes was measured by 2^−ΔΔCt^ method with GAPDH and U6 as the internal control.

### Colony formation assay

Briefly, cultured cells were inoculated into 6-well plates for 14-day incubation, followed by fixation in ethanol. Afterwards, subsequent to staining with crystal violet, cell colonies were subjected to manual counting.

### 5-ethynyl-20-deoxyuridine (EdU) staining assay

Cells were put in 96-well plates at a density of 5 × 10^4^ cells. Cell nucleus was double-stained with EdU and DAPI dye (Beyotime, Shanghai, China). Then cells were observed under the fluorescence microscope for investigation into changes of cell proliferation (Olympus, Tokyo, Japan).

### Wound healing assay

A total of 3 × 10^3^ cells seeded into 6-well plates were subjected to 24-h culture in serum-free medium at 37 °C. When cells reached 80% confluence, using a pipette tip was used to make a straight scratch wound. Then cells were cultured for another 24 h, and scratches were monitored and photographed at 0 and 24 h.

### Transwell assay

In brief, 2 × 10^4^ cells were plated into the upper chambers of transwell plates. Then the bottom chamber was added with 100% complete culture medium. The chambers were added with Matrigel (BD Biosciences San Diego, CA, USA) for invasion assay and no Matrigel for migration assay. After 24 h, cells were stained by crystal violet for counting.

### Subcellular fractionation

Cytoplasmic & Nuclear RNA Purification Kit was applied for isolating and extracting the cytoplasm and nuclear RNA of PCa cells, and then the distribution of MIR99AHG was determined with the help of RT-qPCR analysis. GAPDH and U6 were respectively used as cytoplasmic and nuclear controls.

### Fluorescent in situ hybridization (FISH) and immunofluorescence (IF)

For FISH, subsequent to 15-min fixation with 4% PFA at 37 °C, PANC-1 and SW1990 cells were permeabilized with 0.5% Triton X-100 and hybridized with MIR99AHG probe in buffer, followed by DAPI staining.

For IF, ELAVL1 primary antibody was used to blot overnight at 4℃ and subsequently, blots were incubated with FITC-conjugated secondary antibody. Images were obtained and collected with the utilization of a confocal laser microscope (Olympus).

### RNA immunoprecipitation (RIP)

The EZMagna RIP kit was applied according to the manufacturer’s instructions. A complete RIP cleavage buffer was applied to lyse the cells. The extracts of cells were incubated with coupled magnetic beads conjugated with Anti-Argonaute 2 (Ago2) antibody, ELAVL1 antibody or anti-IgG antibody, followed by incubation at 4℃ for 6 h. Finally, the purified RNA was analyzed by RT-qPCR.

### RNA pull down assay

The RNAs and proteins obtained from cancer cells were mixed with the biotinylated MIR99AHG. Magnetic beads were then added into cells. The pull-downs collected by beads were subject to purification, followed by RT-qPCR and western blot analysis.

### Chromatin immunoprecipitation (ChIP)

PANC-1 and SW1990 cells were subject to 30-min fixation with 1% formaldehyde at room temperature. Sonication was used to cut the DNA to an average size. For chromatin immunoprecipitation, anti-FOXA1 antibody was utilized with anti-IgG as the negative control. Also, subsequent to purification, RT-qPCR analysis was applied for detecting the chromatins.

### Luciferase reporter assay

The MIR99AHG promoter region containing the binding sites (wild type or mutant type) was constructed into the pGL3 vector (Promega, Madison, WI) and co-transfected along with pcDNA3.1/FOXA1 or the empty vector into PCa cells. Similarly, the sequence of MIR99AHG or NOTCH2 mRNA 3’UTR containing wild-type (Wt) and mutant type (Mut) of miR-3129-5p binding sites was inserted into pmirGLO dual-luciferase vector to form pmirGLO-MIR99AHG-Wt/Mut and pmirGLO-NOTCH2-Wt/Mut respectively. Later, we co-transfected miR-3129-5p mimics and NC mimics with the reporter gene into PCa cells. After transfection for 24 h, the cells were collected and lysed. The calculation of luciferase activity was completed with the utilization of Luciferase Reporter Assay system.

### Western blot

The samples were added with RIPA buffer, after homogenation, the total protein was extracted. At room temperature, 5% skimmed dry milk was used to seal the PVDF membranes for 1 h. Subsequent to incubation at 4 °C for a whole night, TBST was applied to wash membrane for 3 times and the secondary antioxidant peroxides were incubated continuously. The antibodies against NOTCH2, HES1, HES6, ELAVL1, HNRNPC and β-actin were all bought from Abcam.

### Tumor xenograft model

BALB/c nude mice (female, 4–6-week-old, 18–20 g) were acquired from Beijing Vital River Laboratory Animal Technology Co. Ltd. PCa cells (1 × 10^6^ per injection) were respectively transfected with indicated plasmids including sh-NC, sh-MIR99AHG#1, sh-MIR99AHG#1 + NC inhibitor, sh-MIR99AHG#1 + miR-3129-5p inhibitor, sh-MIR99AHG#1 + pcDNA3.1 and sh-MIR99AHG#1 + pcDNA3.1/NOTCH2. Then, the transfected cells were implanted into the mice via subcutaneous injection. The measurement of tumor volumes was conducted every 5 days after being apparently observed and calculated with the following formula: Volume = (length × width^2^)/2. After 30 days, all mice were sacrificed. The animal experiments were implemented under the approval of Shengjing Hospital of China Medical University.

### Statistical analysis

SPSS 17.0 were used to conduct statistical analyses and all data were expressed as mean ± SD. ANOVA and Student’s t-test were used to analyze the difference between the two or more groups. It was considered to be statistically significant when P value was less than 0.05. All the assays were performed in triplicate.

## Results

### MIR99AHG advances cell malignant behaviors in PCa

**Fig. 1:**
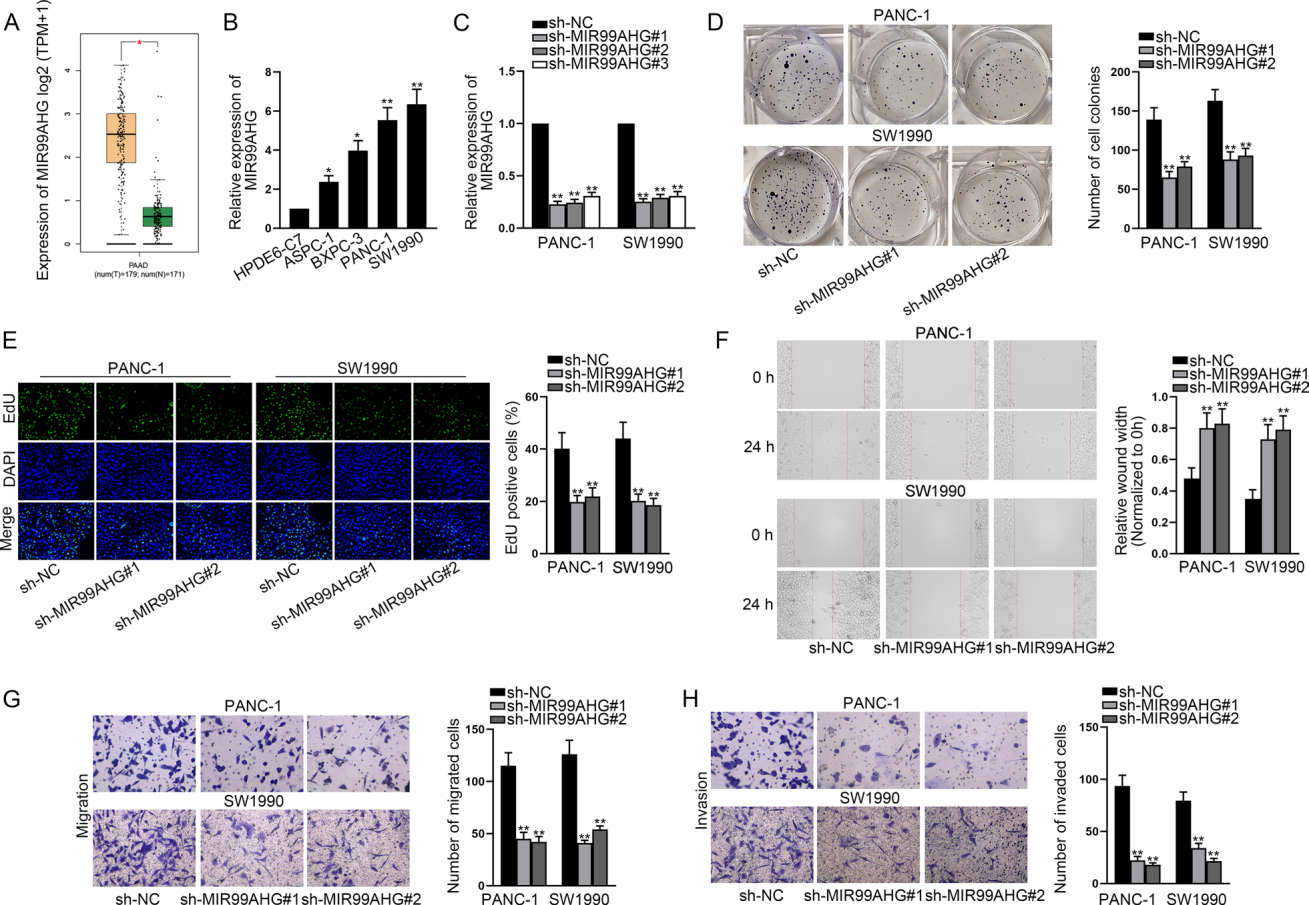
MIR99AHG advances PCa cell malignant phenotype. **A** GEPIA2 database demonstrated MIR99AHG expression in tumor tissues and normal pancreatic tissues. **B** MIR99AHG expression in different cell lines was quantified with the help of RT-qPCR analysis. **C** The measurement of MIR99AHG expression was conducted in PCa cells with the transfection of indicated plasmids (sh-NC and shRNAs targeting MIR99AHG). **D**, **E** Cell proliferation was assessed by implementing colony formation and EdU assays. **F** Wound healing assay examined the migratory capacity of PCa cells. **G**, **H** The observation of migrated or invaded cells and the evaluation of cell migratory and invasive abilities were done with the help of transwell assay. *P < 0.05, **P < 0.01

As exhibited in Fig. 1A, GEPIA2 database (http://gepia2.cancer-pku.cn/#index) displayed that compared with normal pancreatic tissues, MIR99AHG was aberrantly up-regulated in PCa tissues. RT-qPCR also demonstrated that the expression of MIR99AHG was dramatically high in PCa cell lines (ASPC-1, BXPC-3, PANC-1 and SW1990) in comparison with normal pancreatic cell line (HPDE6-C7) (Fig. 1B). To further assess the role of MIR99AHG in PCa cells, MIR99AHG was knocked down in PANC-1 and SW1990 cells (Fig. 1C). Subsequently, colony formation and EdU assays revealed that when MIR99AHG expression was reduced, cell proliferation was accordingly suppressed (Fig. 1D, E). The results of wound healing assays indicated that depletion of MIR99AHG obviously weakened the migratory capacity of PANC-1 and SW1990 cells (Fig. 1F). Moreover, transwell assays were carried out to observe cell migration and invasion. As expected, MIR99AHG deficiency caused the impeded migration and invasion of PCa cells (Fig. 1G, H). To be summarized, MIR99AHG acts as an oncogene in PCa cells.

### FOXA1 serves as a transcription activator of MIR99AHG

**Fig. 2:**
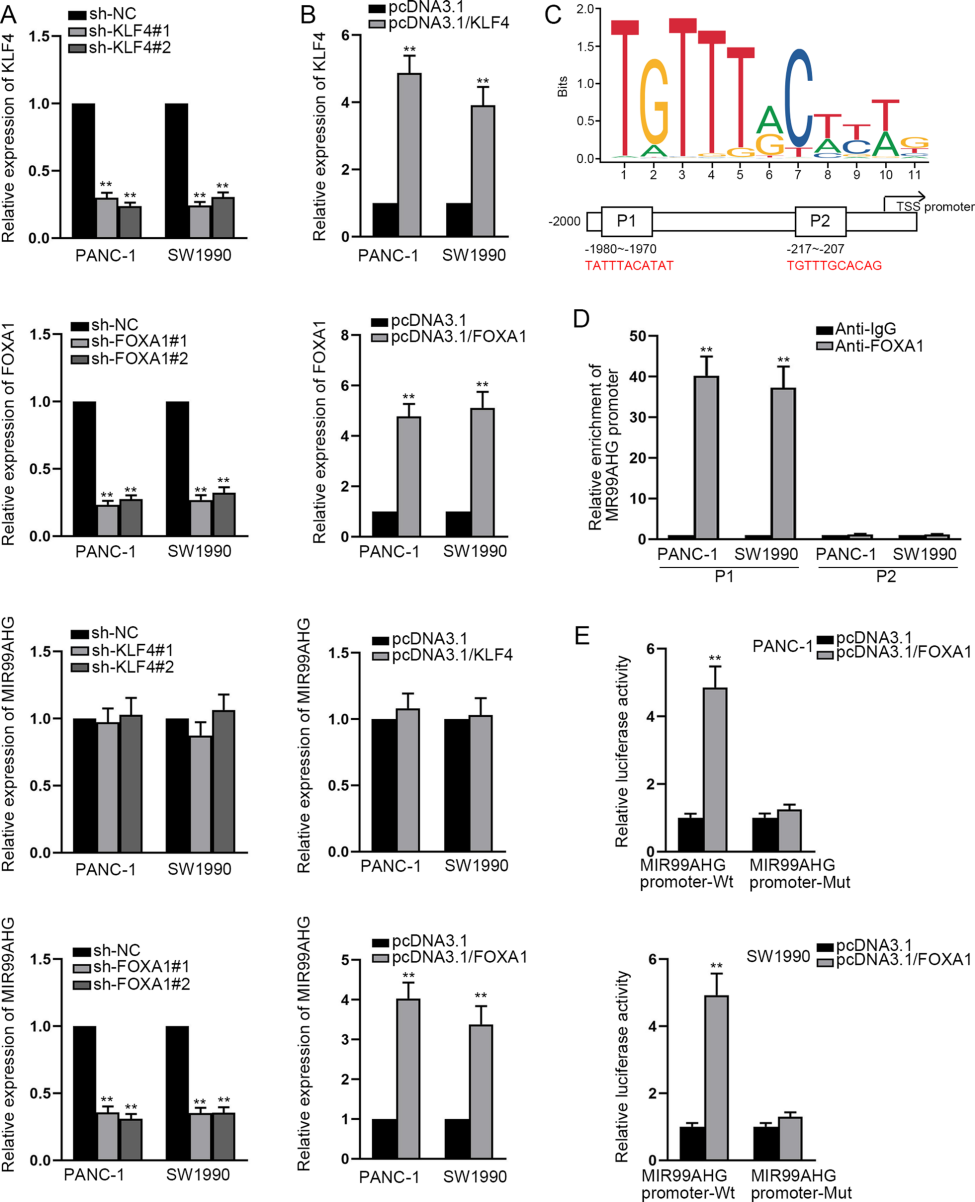
FOXA1 serves as a transcription activator of MIR99AHG. **A**, **B** RT-qPCR detected MIR99AHG expression when KLF4 or FOXA1 was knocked down or overexpressed. **C** JASPAR was applied for prediction of the binding motif of FOXA1 and 2 potential binding sites between FOXA1 and MIR99AHG promoter. **D**, **E** ChIP and luciferase reporter assay validated the binding between FOXA1 and MIR99AHG promoter. **P < 0.01

To explore the biological mechanism underlying high expression of MIR99AHG, HumanTFDB (http://bioinfo.life.hust.edu.cn/HumanTFDB#!/) and GEPIA database (http://gepia.cancer-pku.cn/index.html) were utilized to screen out 2 potential transcription factors of MIR99AHG (KLF4 and FOXA1) (Additional file 1: Fig. 1A). Compared with normal pancreatic tissues, the high expression of KLF4 and FOXA1 in PCa tissues was also exhibited in Additional file 1: Fig. 1B, C. To ascertain the transcription factor, we respectively knocked down KLF4 and FOXA1, discovering that MIR99AHG expression was regulated only by FOXA1 (Fig. 2A). The similar result was observed when KLF4 and FOXA1 were overexpressed (Fig. 2B). Hence, FOXA1 was chosen for the following studies. The binding motif of FOXA1 and predicted 2 binding sites between FOXA1 and MIR99AHG promoter were shown in Fig. 2C. The experimental results of ChIP assay suggested that FOXA1 bound with the P1 region of the MIR99AHG promoter (Fig. 2D). Besides, luciferase reporter assay uncovered that FOXA1 up-regulation led to a dramatic increase of luciferase activity in wild-type MIR99AHG promoter, which further determined the function of FOXA1-binding sites (Fig. 2E). Taken together, FOXA1 induces the up-regulation of MIR99AHG in PCa cells.

### MIR99AHG directly interacts with miR-3129-5p

**Fig. 3:**
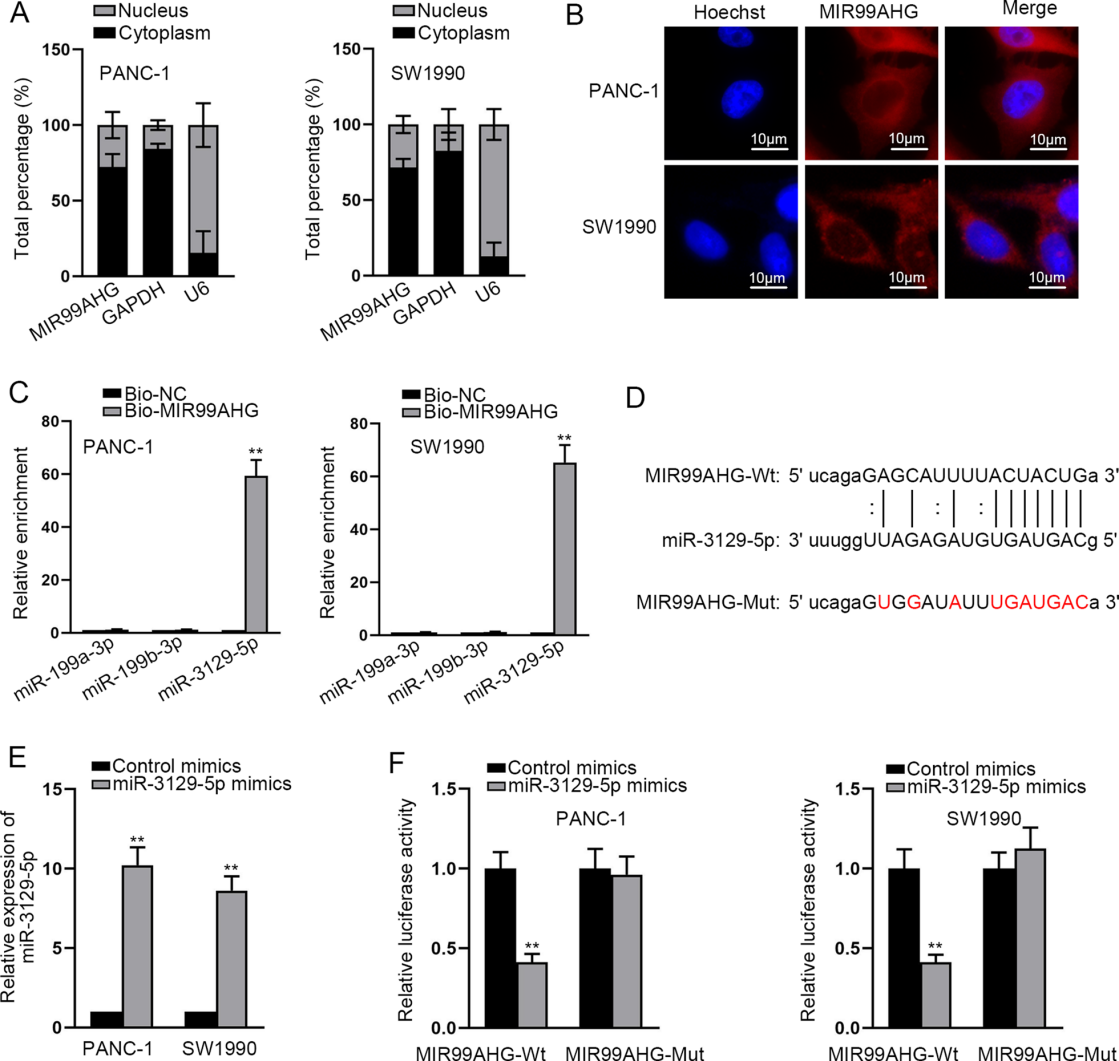
MIR99AHG directly interacts with miR-3129-5p. **A**, **B** The accumulation of MIR99AHG in PCa cells was explored by subcellular fractionation and FISH assays. **C** RNA-RNA pull down assay displayed the enrichment of miR-3129-5p, miR-199a-3p and miR-199b-3p by Bio-MIR99AHG. **D** The binding sequence between MIR99AHG and miR-3129-5p, predicted on starBase, was demonstrated. **E** The detection and analysis of miR-3129-5p expression was done in miR-3129-5p mimics-transfected cells. **F** Luciferase reporter assay was done for verifying the interaction between MIR99AHG and miR-3129-5p. **P < 0.01

To determine the subcellular location of MIR99AHG in PCa cells, we implemented subcellular fractionation and FISH assays. It was manifested from the experimental results that the prominent distribution of MIR99AHG was in the cytoplasm of PANC-1 and SW1990 cells (Fig. 3A, B). Hence, we conjectured that MIR99AHG might function as a competing endogenous RNA (ceRNA) by interacting with miRNAs. We chose 3 miRNAs (miR-199a-3p, miR-199b-3p and miR-3129-5p) based on starBase (Degradome Data ≥ 1) (http://starbase.sysu.edu.cn/index.php). After silencing or overexpressing MIR99AHG, relative expression of the three miRNAs was detected and the results showed their expression had no obvious change (Additional file 1: Fig. 1D, E). Based on RNA pull down assay, miR-3129-5p had a strong affinity with MIR99AHG and was identified as the potential downstream of MIR99AHG (Fig. 3C). The binding sequence between MIR99AHG and miR-3129-5p was displayed in Fig. 3D. PANC-1 and SW1990 cells were transfected with miR-3129-5p mimics to enhance the expression of miR-3129-5p (Fig. 3E). Luciferase reporter assay pointed out that the transfection of miR-3129-5p mimics resulted in the significant reduction in the luciferase activity of the MIR99AHG-Wt (Fig. 3F). In conclusion, miR-3129-5p is sponged by MIR99AHG in PCa cells.

### MIR99AHG is related to the activation of Notch signaling pathway

**Fig. 4:**
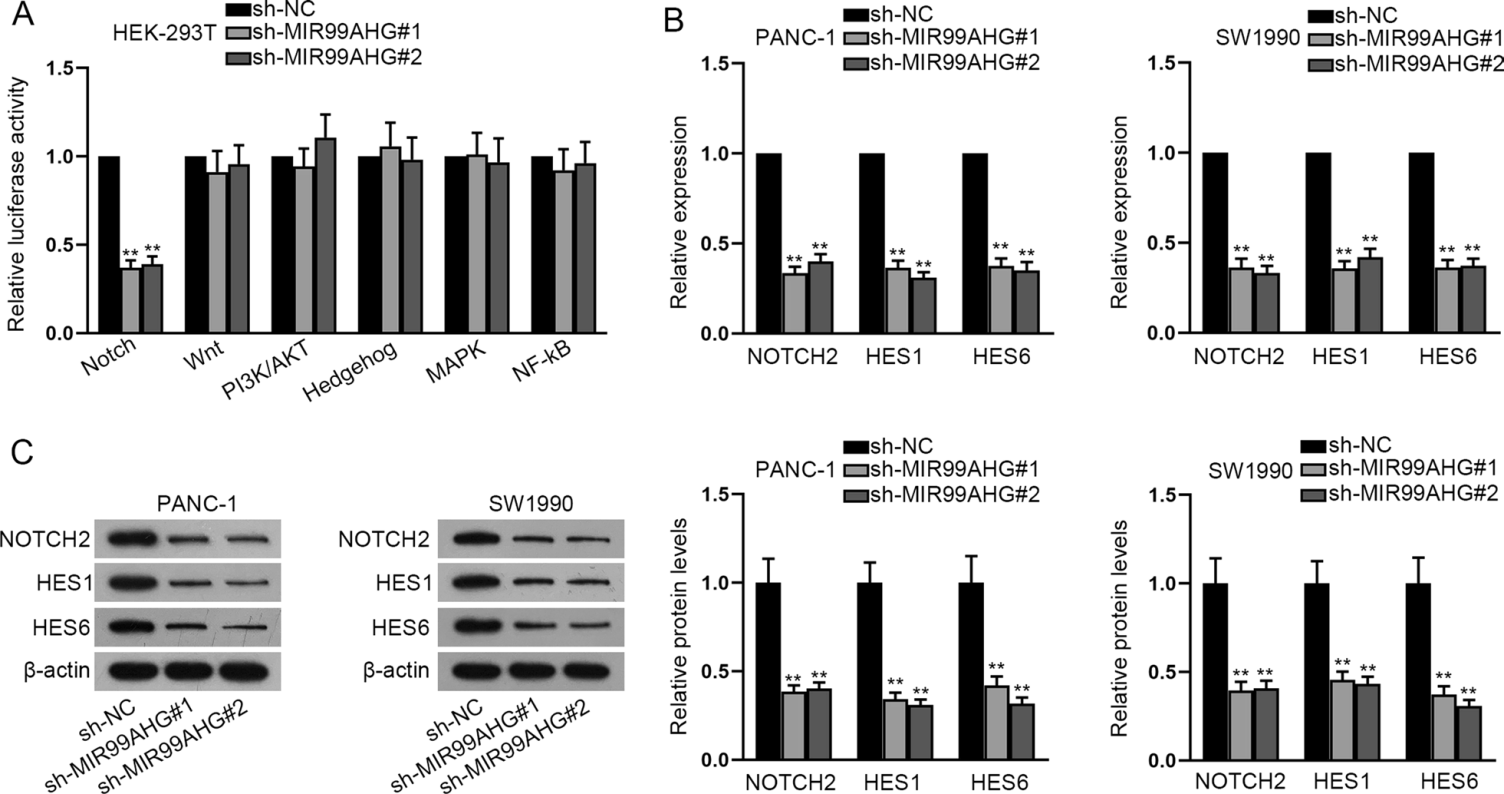
MIR99AHG is related to the activation of Notch signaling pathway. **A** Luciferase reporter assay was applied for testing the luciferase activity of indicated signaling pathways when MIR99AHG was silenced. **B**, **C** The measurement of key genes (NOTCH2, HES1 and HES6) was done with the utilization of RT-qPCR and western blot analysis in response to the decline in MIR99AHG level. **P < 0.01

Luciferase reporter assay intriguingly revealed that MIR99AHG knockdown restrained the luciferase activity in Notch signaling pathway (Fig. 4A). From the experimental results of RT-qPCR and western blot analysis, we observed that the expression of key factors of Notch signaling pathway (NOTCH2, HES1 and HES6) was dramatically mitigated after MIR99AHG inhibition (Fig. 4B, C). All these findings verify that MIR99AHG activates the Notch signaling pathway.

### NOTCH2 is targeted by miR-3129-5p

**Fig. 5:**
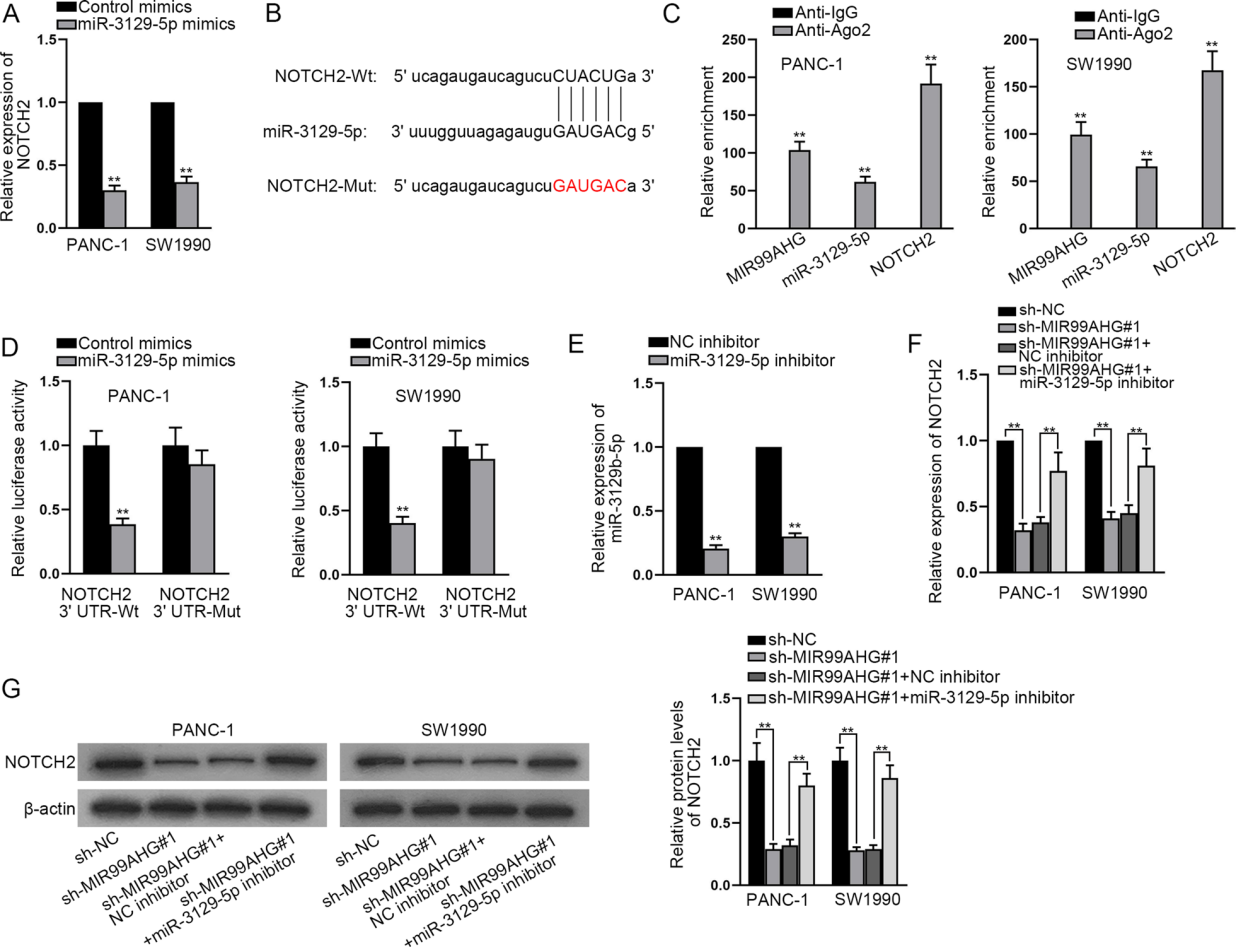
NOTCH2 is targeted by miR-3129-5p. **A** RT-qPCR was applied for measuring NOTCH2 expression after transfection of miR-3129-5p mimics. **B** The binding region between miR-3129-5p and NOTCH2 was displayed. **C** The relationship among MIR99AHG, miR-3129-5p and NOTCH2 was examined by RIP assay. **D** Luciferase reporter assay was done for the detection of the luciferase activity in the NOTCH2 3’UTR-Wt group and NOTCH2 3’UTR-Mut group after transfection of miR-3129-5p mimics. **E** MiR-3129-5p inhibitor was transfected to cut down miR-3129-5p expression. **F**, **G** The detection of NOTCH2 expression was done by means of RT-qPCR and western blot in cells after transfection with indicated plasmids, including sh-NC, sh-MIR99AHG#1, sh-MIR99AHG#1 + NC inhibitor and sh-MIR99AHG#1 + miR-3129-5p inhibitor. **P < 0.01

It is commonly acknowledged that NOTCH2 is a critical participator in the Notch signaling pathway. GEPIA database detected the expression of NOTCH2 in PCa tissues and as shown in Additional file 1: Fig. 1F, NOTCH2 displayed a high expression in PCa tissues. Through RT-qPCR analysis, we found that NOTCH2 expression was cut down after transfection of miR-3129-5p mimics (Fig. 5A). In order to confirm NOTCH2 was the target gene of miR-3129-5p, we conducted further analysis. The binding region between miR-3129-5p and NOTCH2 was predicted through starBase and demonstrated in Fig. 5B. Moreover, RIP assay confirmed the interaction among MIR99AHG, miR-3129-5p and NOTCH2 (Fig. 5C). The binding between miR-3129-5p and NOTCH2 was verified by luciferase reporter assay (Fig. 5D). After transfection of miR-3129-5p inhibitor, miR-3129-5p expression was lessened (Fig. 5E). RT-qPCR and western blot analysis disclosed that knockdown of MIR99AHG significantly reduced NOTCH2 expression in both PANC-1 and SW1990 cell lines, while miR-3129-5p inhibitor partially restored NOTCH2 expression (Fig. 5F, G). In a word, NOTCH2 is a target gene of miR-3129-5p.

### MIR99AHG recruits ELAVL1 protein to stabilize NOTCH2 mRNA

**Fig. 6:**
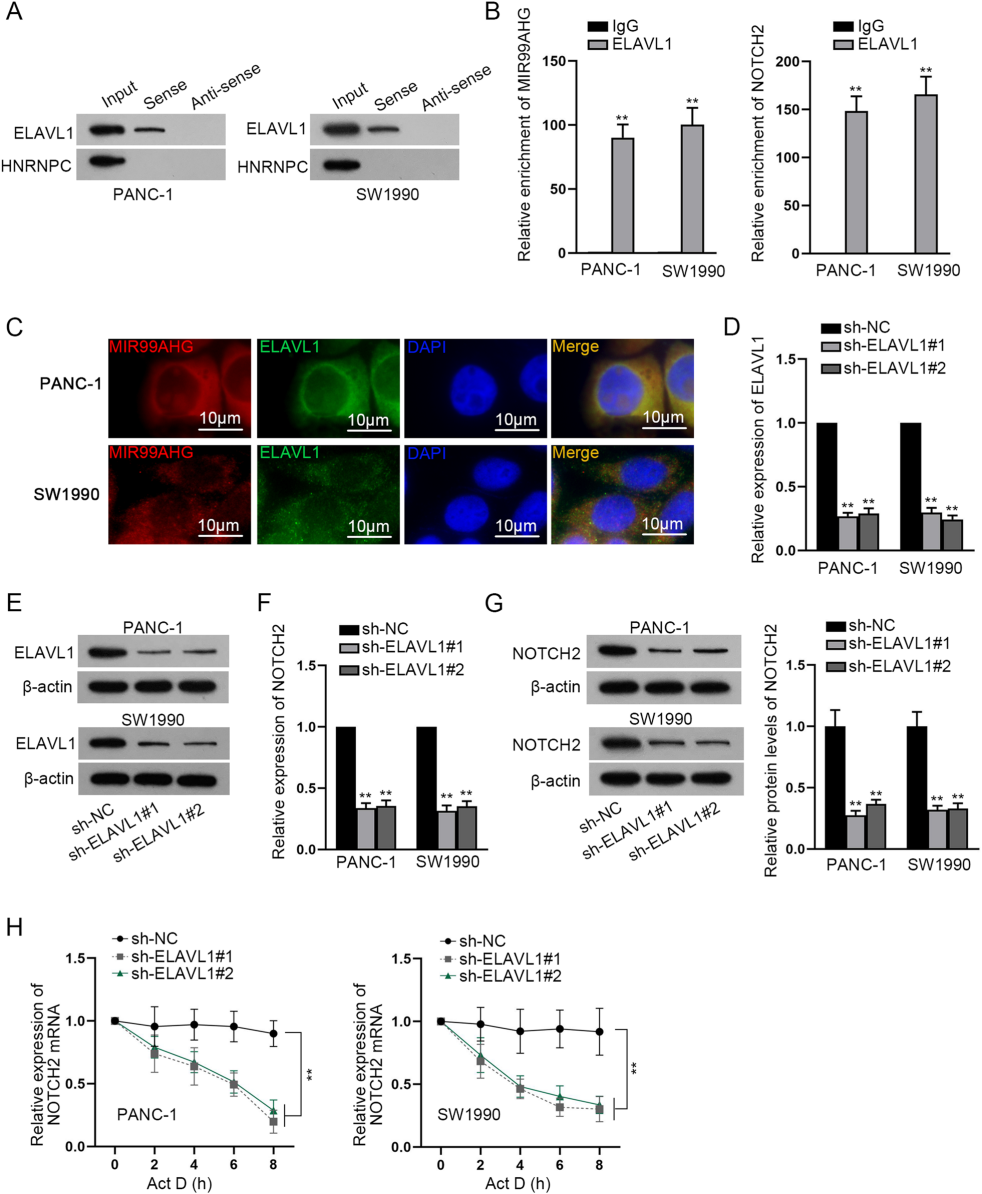
MIR99AHG recruits ELAVL1 protein to stabilize NOTCH2 mRNA. **A**, **B** RNA pull down and RIP assays exhibited the affinity between MIR99AHG and ELAVL1. **C** The co-localization of MIR99AHG and ELAVL1 was manifested by FISH and immunofluorescence assays. **D**, **E** RT-qPCR and western blot were applied for detecting and analyzing ELAVL1 expression in PCa cells after the transfection of indicated plasmids. **F**, **G** The detection of NOTCH2 expression was done by the utilization of RT-qPCR and western blot after ELAVL1 was down-regulated. **H** After adding Act D, the stability of NOTCH2 mRNA was tested. **P < 0.01

In view of the finding that down-regulation of miR-3129-5p had no capacity to completely restore the reduced NOTCH2 expression caused by silencing of MIR99AHG, we speculated that another common mechanism, RNA binding protein (RBP) mechanism also existed. Based on GEPIA and starBase, 2 RBPs (ELAVL1 and HNRNPC) were screened out (Additional file 2: Fig. 2A). From RNA pull down assay, ELAVL1 was chosen for the subsequent study on account of its strong binding with MIR99AHG in PCa cells (Fig. 6A). The interaction between MIR99AHG and ELAVL1, ELAVL1 and NOTCH2 was also confirmed by RIP assay (Fig. 6B). The co-localization of MIR99AHG and ELAVL1 in cytoplasm of PANC-1 and SW1990 cells was uncovered based on the experimental results of FISH and IF assays (Fig. 6C). In response to the transfection of sh-ELAVL1#1/2 plasmids, ELAVL1 expression was overtly reduced (Fig. 6D, E). Moreover, NOTCH2 expression was also decreased owing to ELAVL1 deficiency (Fig. 6F, G). Furthermore, down-regulation of ELAVL1 also lowered the stability of NOTCH2 mRNA after Actinomycin D (Act D) was added (Fig. 6H). ELAVL1 expression was detected in sh-MIR99AHG#1-transfected PCa cells and it was indicated that MIR99AHG silence did not affect the expression of ELAVL1 (Additional file 2: Fig. 2B). Then RIP assay was conducted after MIR99AHG down-regulation to test enrichment of MIR99AHG and NOTCH2 in anti-ELAVL1. After MIR99AHG inhibition, less MIR99AHG or NOTCH2 could be precipitated by anti-ELAVL1 (Additional file 2: Fig. 2C). ELAVL1 overexpression efficiency was determined through RT-qPCR (Additional file 2: Fig. 2D). Then, expression of NOTCH2 mRNA was detected after MIR99AHG inhibition and the results showed NOTCH2 expression declined in response to MIR99AHG depletion, and the declined NOTCH2 expression could not be rescued by ELAVL1 augment (Additional file 2: Fig. 2E). In addition, down-regulation of MIR99AHG reduced the stability of NOTCH2 mRNA after Act D treatment, and co-transfection of pcDNA3.1/ELAVL1 could hardly reverse the condition (Additional file 2: Fig. 2F). Taken together, MIR99AHG recruits ELAVL1 protein to stabilize NOTCH2 mRNA in PCa cells.

### Down-regulation of miR-3129-5p or up-regulation of NOTCH2 rescues the abrogated PCa cell malignant behaviors imposed by MIR99AHG knockdown

**Fig. 7:**
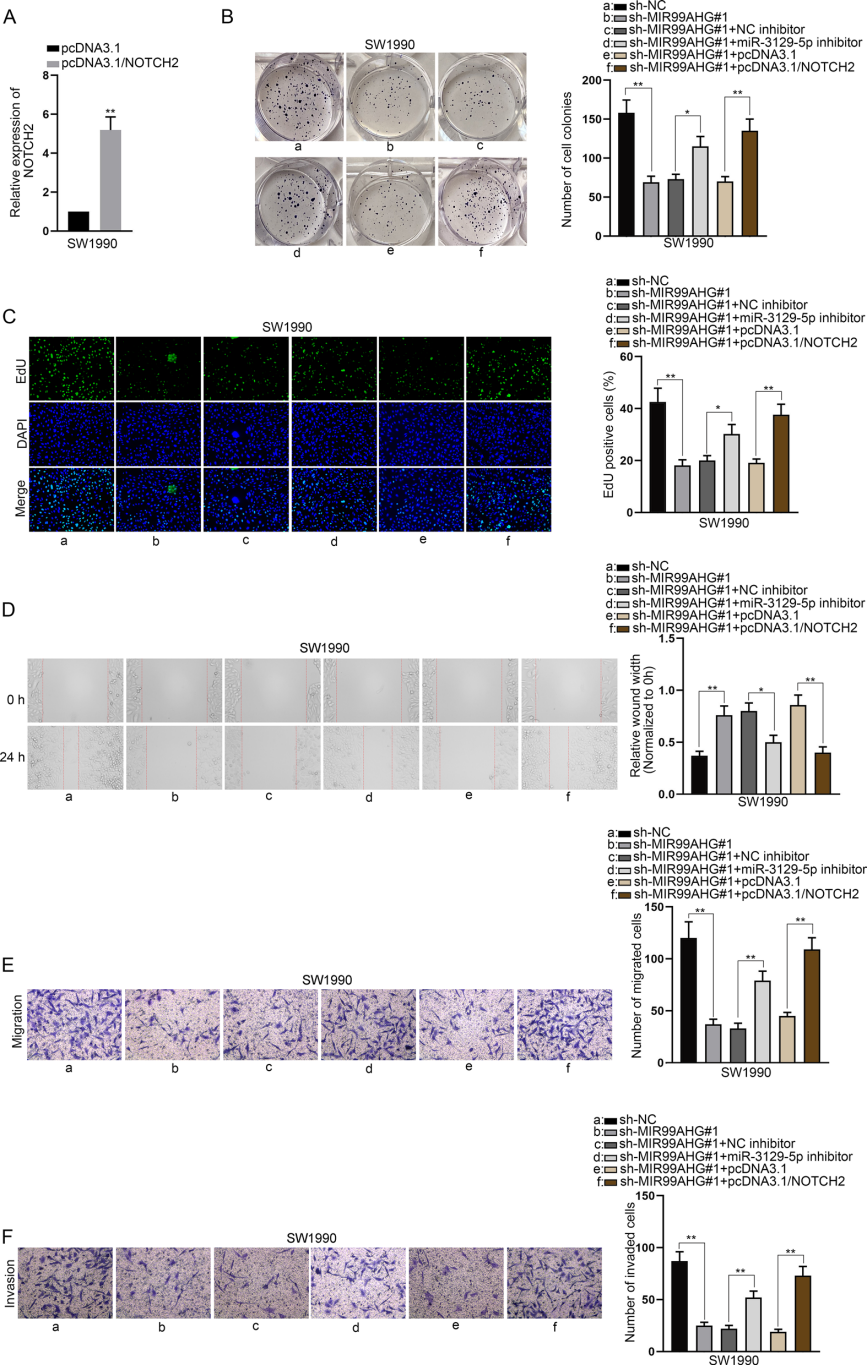
Down-regulation of miR-3129-5p or up-regulation of NOTCH2 rescues the abrogated PCa cell malignant behaviors imposed by MIR99AHG knockdown. **A** NOTCH2 was overexpressed in SW1990 cells. **B**, **C** Cell proliferation was observed in SW1990 cells with the transfection of indicated plasmids including sh-NC, sh-MIR99AHG#1, sh-MIR99AHG#1 + NC inhibitor, sh-MIR99AHG#1 + miR-3129-5p inhibitor, sh-MIR99AHG#1 + pcDNA3.1 and sh-MIR99AHG#1 + pcDNA3.1/NOTCH2 through colony formation assay and EdU assay. **D**, **E** The evaluation of cell migration in different transfection conditions was done by means of wound healing and transwell assays. **F** Transwell assays were applied to conduct investigation in the invasive capacity of SW1990 cells in different groups. *P < 0.05, **P < 0.01

Relative expression of NOTCH2, miR-3129-5p, ELAVL1 and FOXA1 was detected in PCa cell lines and normal pancreatic cell line (HPDE6-C7). It was learnt from the data that only miR-3129-5p was down-regulated in PCa cell lines compared with HPDE6-C7 while other three genes were all up-regulated in PCa cell lines (Additional file 3: Fig. 3A–D). Before implementation of rescue assays, NOTCH2 was overexpressed in SW1990 cells (Fig. 7A). Cell proliferation assays indicated that miR-3129-5p depletion partially restored the weakened proliferative capacity of SW1990 cells caused by MIR99AHG silencing while NOTCH2 up-regulation completely rescued this effect (Fig. 7B, C). Wound healing and transwell assays both suggested that MIR99AHG inhibition induced suppressed cell migration was partially offset by miR-3129-5p inhibitor while being completely countervailed by NOTCH2 overexpression (Fig. 7D, E). Besides, from the experimental results of transwell assay, we observed that MIR99AHG reduction hindered cell invasion while miR-3129-5p deficiency partially counteracted this effect and NOTCH2 up-regulation completely restored this effect (Fig. 7F). Above results all validate the MIR99AHG/miR-3129-5p/NOTCH2 axis in PCa cells.

### MIR99AHG facilitates PCa progression by elevating NOTCH2 expression

In vivo experiments were then carried out to further certify our conclusion. Mice were injected with PCa cells transfected with different plasmids and the tumor volume and weight of mice in different groups were analyzed. The results showed that NOTCH2 overexpression could recover the decreased tumor volume and weight caused by MIR99AHG inhibition (Additional file 4: Fig. 4A, B). Above results all validate that MIR99AHG facilitates PCa progression by elevating NOTCH2 expression.

## Discussion

In recent years, the correlation between lncRNAs and cancers has been widely explored [6]. In PCa, the vital roles of lncRNAs have also been proved [10]. MIR99AHG has been deeply investigated in gastric cancer and the results disclose the oncogenic role of MIR99AHG in gastric cancer. Likewise, through bioinformatics analysis and RT-qPCR, we discovered that MIR99AHG was up-regulated in PCa tissues and cells. After a series of functional assays, we concluded that knockdown of MIR99AHG obviously hindered PCa cell proliferation, migration and invasion.

In this study, we found 2 potential transcription factors of MIR99AHG, namely, KLF4 and FOXA1, which were both up-regulated in PCa tissues. Through RT-qPCR, we discovered that only FOXA1 could regulate MIR99AHG. FOXA1 is a transcription factor which has important functions in cancer development [17]. Zhang et al. have demonstrated that FOXA1 mediated lncRNA DSCAM-AS1 contributes to the cancer process via a positive feedback loop [18]. Through our investigation, we discovered that FOXA1 induced the up-regulation of MIR99AHG in PCa cells and had a strong affinity with the promoter of MIR99AHG.

A large amount of studies have attested that lncRNA associated ceRNA regulatory mechanism makes a difference in the pathogenesis of PCa [19]. Through subcellular fractionation and FISH assays, MIR99AHG was found to be prominently distributed in the cytoplasm of PCa cells, which suggested that MIR99AHG might act as a ceRNA by serving as a sponge for miRNAs. According to bioinformatics analysis, three miRNAs were predicted to be potential target miRNA of MIR99AHG. Then, miR-3129-5p was selected out through RIP assay. MiR-3129-5p has been reported to be implicated in adolescent obesity and hepatocellular carcinoma [20, 21]. Cao et al. have proposed that miR-3129-5p is sponged by lncRNA MALAT1 and regulates Nova1 expression to influence doxorubicin resistance in hepatocellular carcinoma [22]. Similarly, after luciferase reporter assay, we discovered that miR-3129-5p interacted with MIR99AHG and participated in the progression of PCa.

Then, luciferase reporter assay was conducted in common signaling pathways and only luciferase activity of Notch signaling pathway was decreased after MIR99AHG inhibition. Notch signaling pathway occupies an important position in the development of PCa [23]. Among the determinants of the Notch signaling pathway, NOTCH2 receptor has been reported to exert crucial functions in the carcinogenesis of PCa [24]. Moreover, depletion of NOTCH2 hampers the process of PCa [25]. The expression of three key genes of Notch pathway was tested via western blot and RT-qPCR, showing MIR99AHG inhibition would result in decrease of Notch key gene expression. It indicated that MIR99AHG could activate Notch signaling pathway. Then we explored the relationship between miR-3129-5p and NOTCH2, one of the key genes of Notch signaling pathway. It was learnt from RIP, luciferase reporter assay and rescue experiments that MIR99AHG stimulated Notch signaling pathway by sponging miR-3129-5p and targeting NOTCH2.

For the reason that miR-3129-5p down-regulation couldn’t fully restore the reduced expression of NOTCH2 due to MIR99AHG deficiency, we speculated that there existed another common regulatory mechanism. ELAVL1, which is also called HuR, has been widely investigated in many cancers as an RBP. Specifically, lncRNA HOXB-AS1 has been testified to recruit ELAVL1 and thereby maintain FUT4 mRNA stability in multiple myeloma [26]. Moreover, the involvement of B4GALT1-AS1 in cell stemness and migration in osteosarcoma by recruiting HuR has also been suggested in a past study [27]. Additionally, EGFR-AS1 binds with HuR to mediate EGFR mRNA stability [28]. In our study, we discovered that ELAVL1 was recruited by MIR99AHG and could stabilize NOTCH2 mRNA.

Eventually, rescue assays proved that MIR99AHG depletion abrogated PCa cell proliferation, migration and invasion while miR-3129-5p down-regulation partially restored this effect and NOTCH2 up-regulation completely rescued this effect. In addition, in vivo experiments also confirmed MIR99AHG could promote PCa progression by elevating NOTCH2 expression.

In summary, MIR99AHG expression was distinctly high in PCa tissues and cells. Deficiency of MIR99AHG impeded cell proliferation, migration, and invasion. Additionally, FOXA1 induced MIR99AHG enhanced NOTCH2 expression by interacting with miR-3129-5p and ELAVL1 protein, thereby getting involved in the development of PCa through the activation of Notch signaling pathway. All these findings indicated that MIR99AHG might be considered as a potential biomarker for PCa diagnosis, treatment and prognosis.

## Conclusion

Altogether, the investigation of FOXA1/MIR99AHG/miR-3129-5p/ELAVL1/NOTCH2 axis in the progression of PCa might provide a meaningful revelation for PCa diagnosis and treatment.

## Supplementary Information

**Additional file 1**: **Figure S1**. (A) Transcriptional factors KLF4 and FOXA1 were screened out based on HumanTFDB and GEPIA database. (B-C) GEPIA database demonstrated KLF4 and FOXA1 expression in tumor tissues and normal pancreatic tissues. (D-E) Expression of miRNAs after MIR99AHG depletion or augment was tested by RT-qPCR. (F) GEPIA database demonstrated NOTCH2 expression in tumor tissues and normal pancreatic tissues. *P < 0.05.

**Additional file 2**: **Figure S2**. (A) 2 potential RBPs were selected out by GEPIA and starBase. (B) Expression of ELAVL1 in sh-MIR99AHG#1-transfected cells was detected and analyzed via RT-qPCR and western blot. (C) In RIP assays, the RNAs enriched by anti-ELAVL1 were subjected to RT-qPCR analysis. (D) ELAVL1 level was tested in pcDNA3.1/ELAVL1 transfected cells by means of RT-qPCR. (E) NOTCH2 mRNA expression was analyzed in cells with the transfection of indicated plasmids via RT-qPCR. (F) After adding Act D, the stability of NOTCH2 mRNA was tested. **P < 0.01.

**Additional file 3**: **Figure S3**. (A-D) Relative expression of NOTCH2, miR-3129-5p, ELAVL1 and FOXA1 was detected in different cell lines. *P < 0.05, **P < 0.01.

**Additional file 4**: **Figure S4**. (A) Images of tumors excised from mice were presented. (B) Analysis of tumor growth was conducted. **P < 0.01.

## Data Availability

Not applicable.

## Abbreviations

PCa: Pancreatic cancer
lncRNAs: Long non-coding RNAs
MIR99AHG: Mir-99a-let-7c cluster host gene
FOXA1: Forkhead box A1
NOTCH2: Notch receptor 2
miR-3129-5p: MicroRNA-3129-5p
ELAVL1: ELAV like RNA binding protein 1
ATCC: American Type Culture Collection
DMEM: Dulbecco’s Modified Eagle’s Medium
shRNAs: Short hairpin RNAs
RT-qPCR: Quantitative real-time PCR
EdU: 5-Ethynyl-20-deoxyuridine
FISH: Fluorescent in situ hybridization
IF: Immunofluorescence
RIP: RNA immunoprecipitation
Ago2: Argonaute 2
ChIP: Chromatin immunoprecipitation
Wt: Wild-type
Mut: Mutant type
Act D: Actinomycin D
ceRNA: Competing endogenous RNA
RBP: RNA binding protein
