# Supplementary figures and images for: LncRNA MIR99AHG mediated by FOXA1 modulates NOTCH2/Notch signaling pathway to accelerate pancreatic cancer through sponging miR-3129-5p and recruiting ELAVL1
Source: Cancer Cell Int. 2021 Dec 15;21:674. doi: 10.1186/s12935-021-02189-z (PMC8675481; doi:10.1186/s12935-021-02189-z)

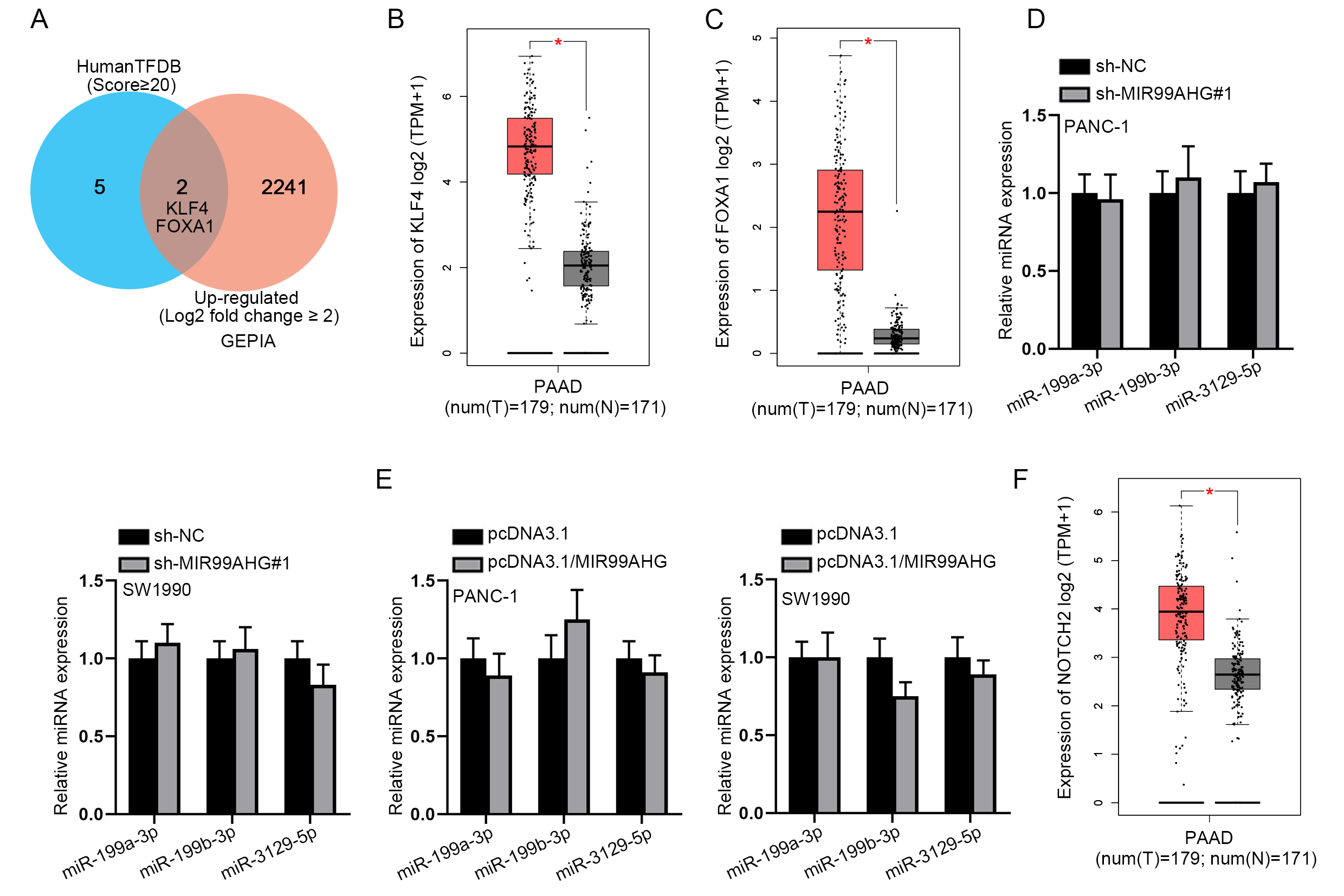

Supplement: Supplementary file 1 — Additional file 1: Figure S1. (A) Transcriptional factors KLF4 and FOXA1 were screened out based on HumanTFDB and GEPIA database. (B-C) GEPIA database demonstrated KLF4 and FOXA1 expression in tumor tissues and normal pancreatic tissues. (D-E) Expression of miRNAs after MIR99AHG depletion or augment was tested by RT-qPCR. (F) GEPIA database demonstrated NOTCH2 expression in tumor tissues and normal pancreatic tissues. *P < 0.05. [file 12935_2021_2189_MOESM1_ESM.tif]

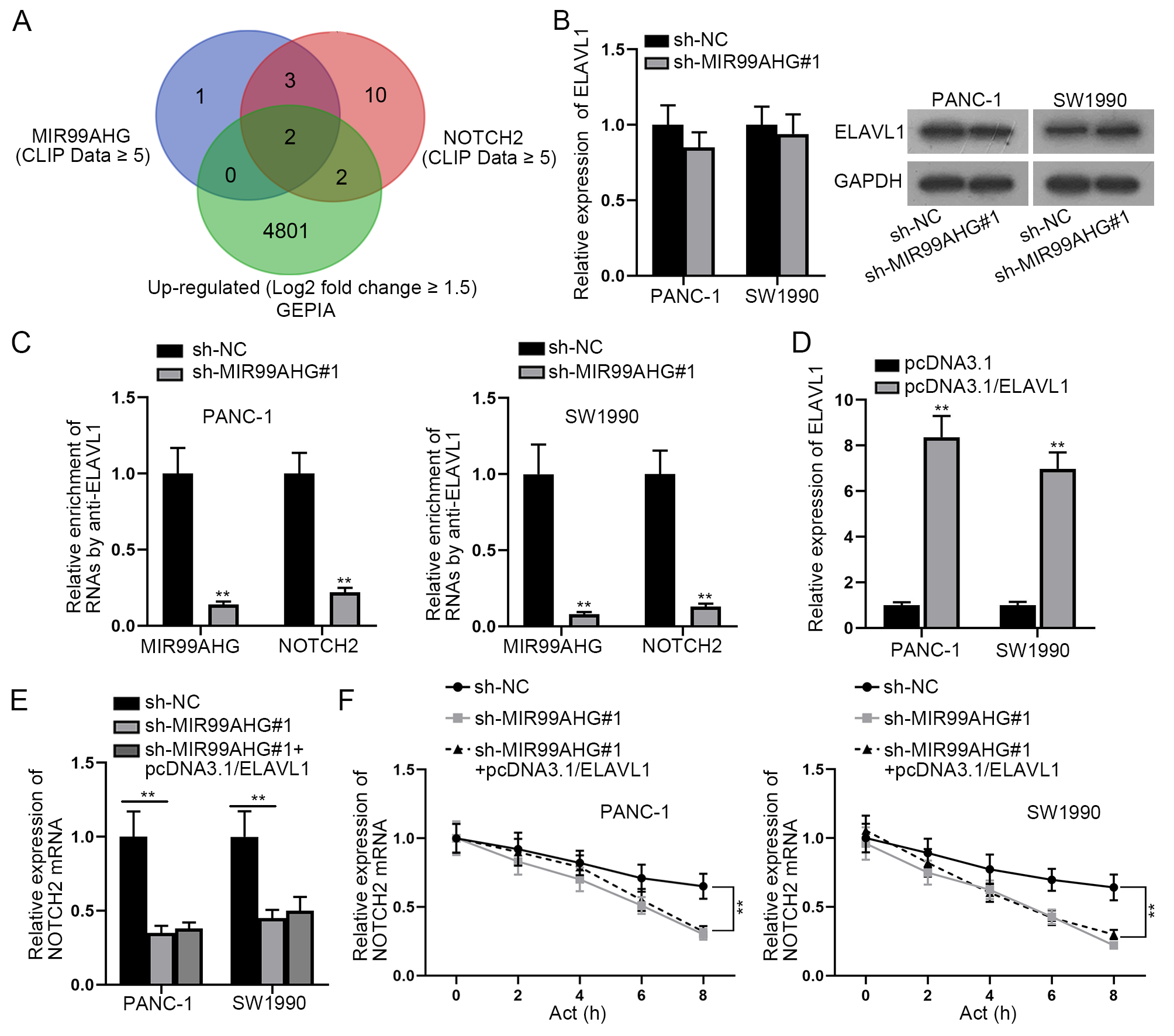

Supplement: Supplementary file 2 — Additional file 2: Figure S2. (A) 2 potential RBPs were selected out by GEPIA and starBase. (B) Expression of ELAVL1 in sh-MIR99AHG#1-transfected cells was detected and analyzed via RT-qPCR and western blot. (C) In RIP assays, the RNAs enriched by anti-ELAVL1 were subjected to RT-qPCR analysis. (D) ELAVL1 level was tested in pcDNA3.1/ELAVL1 transfected cells by means of RT-qPCR. (E) NOTCH2 mRNA expression was analyzed in cells with the transfection of indicated plasmids via RT-qPCR. (F) After adding Act D, the stability of NOTCH2 mRNA was tested. **P < 0.01. [file 12935_2021_2189_MOESM2_ESM.tif]

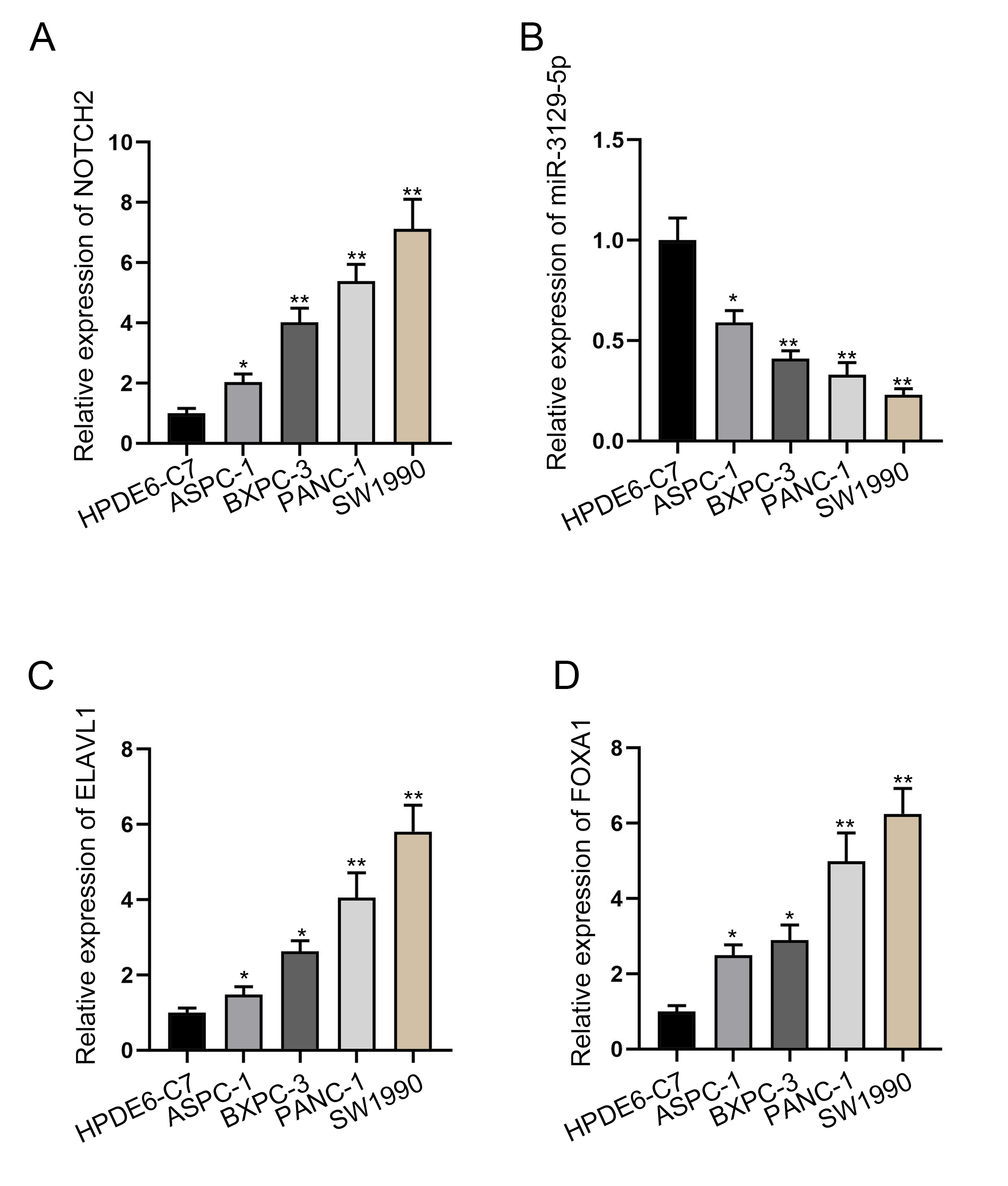

Supplement: Supplementary file 3 — Additional file 3: Figure S3. (A-D) Relative expression of NOTCH2, miR-3129-5p, ELAVL1 and FOXA1 was detected in different cell lines. *P < 0.05, **P < 0.01. [file 12935_2021_2189_MOESM3_ESM.tif]

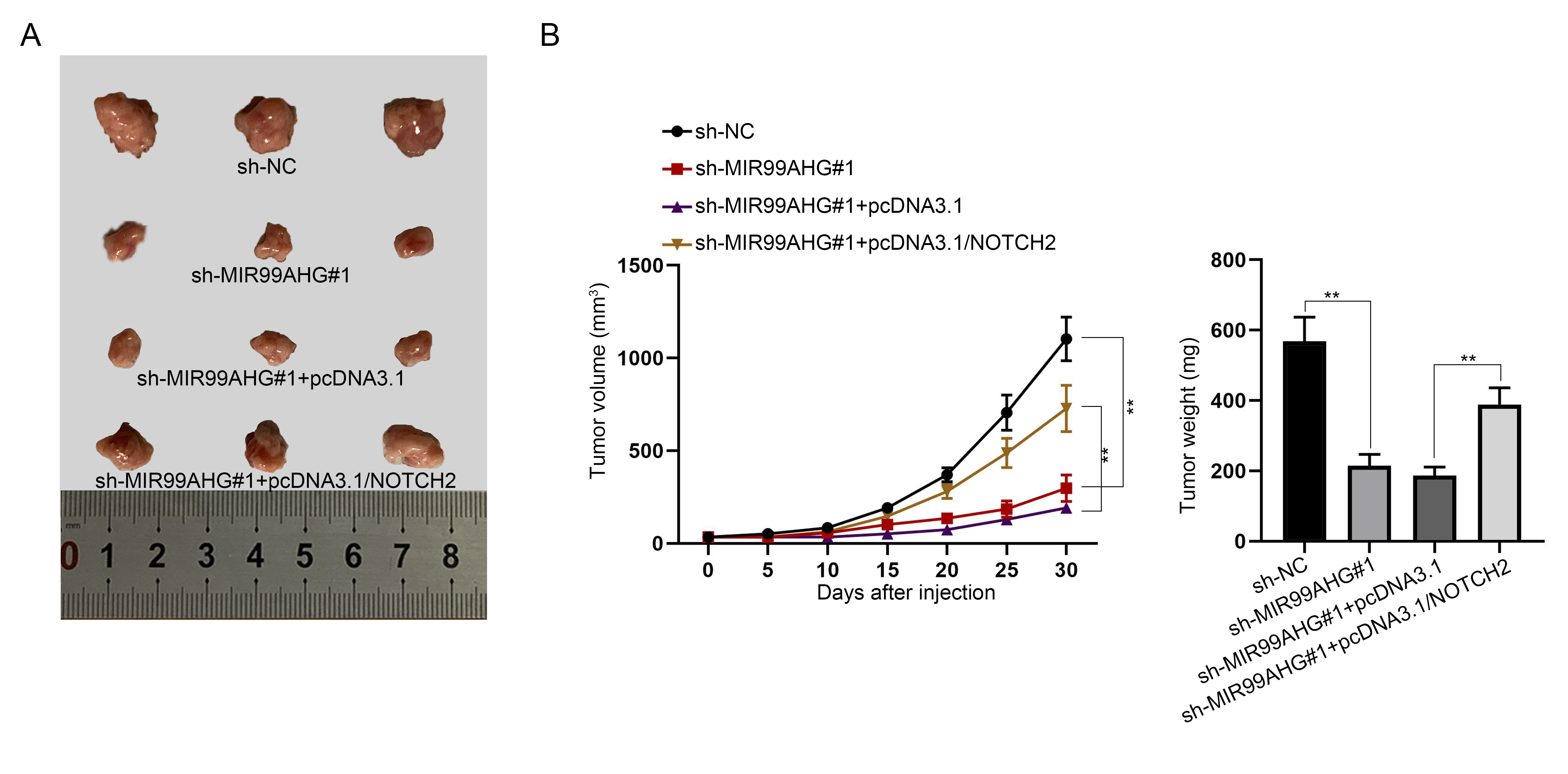

Supplement: Supplementary file 4 — Additional file 4: Figure S4. (A) Images of tumors excised from mice were presented. (B) Analysis of tumor growth was conducted. **P < 0.01. [file 12935_2021_2189_MOESM4_ESM.tif]
